# Evidence for population expansion of Cotton pink bollworm *Pectinophora gossypiella* (Saunders) (Lepidoptera: Gelechiidae) in India

**DOI:** 10.1038/s41598-020-61389-1

**Published:** 2020-03-16

**Authors:** V. Chinna Babu Naik, Pratik P. Pusadkar, Sandesh T. Waghmare, Raghavendra K. P., Sandhya Kranthi, Sujit Kumbhare, V. S. Nagrare, Rishi Kumar, Tenguri Prabhulinga, Nandini Gokte-Narkhedkar, V. N. Waghmare

**Affiliations:** 1 ICAR-Central Institute for Cotton Research. Nagpur, Maharashtra, India; 2 ICAR-Central Institute for Cotton Research, Regional station, Sirsa, India

**Keywords:** Evolution, Plant sciences

## Abstract

Pink bollworm, *Pectinophora gossypiella* (Saunders) infestation on *Bt* cotton is a major concern to cotton production in India. The genetic diversity and phylogeographic structure of the insect in light of PBW resistance needs to be revisited. The objective of this study was to identify different haplotypes of pink bollworm and their distribution in India. To achieve this we studied the population structure in 44 cotton growing districts of India. The partial mitochondrial COI sequence analyses of 214 pink bollworm populations collected from 44 geographical locations representing 9 cotton growing states of India were analysed. Genetic diversity analysis exhibited presence of 27 haplotypes, among them Pg_H1 and Pg_H2 were the most common and were present in 143 and 32 populations, respectively. Distributions of pairwise differences obtained with partial COI gene data from the overall Indian populations are unimodal, suggesting population expansion in India. Significant neutrality test on the basis of Tajima’ D and Fu’s Fs presented a star-shaped haplotype network together with multiple haplotypes. The unimodal mismatch distribution, rejection of neutrality test with significant negative values supported the theory of demographic expansion in cotton pink bollworm populations in India. Genetic data not only provides us with a perspective of population genetics, but also that the two populations of pink bollworm, those occurring early in the season are genetically close to the late season populations with respect to their partial CO1 region. Resistance to Cry toxins does not seem to have had an impact on this region of the mt DNA in populations of pink bollworm.

## Introduction

Cotton is one of world’s most essential fiber crops having global significance, cultivated in tropical and subtropical regions of more than 70 countries^1,2^. In India, cotton is mainly cultivated in three distinct agro-ecological zones (North, Central and South)^3,4^. Cotton is cultivated on an area of 12.43 million ha with average productivity of 505.46 kg/ha in India during 2017–18^5^. Several biotic and abiotic stresses are constraints in cotton production including the bollworm complex and sucking pests^6^. Among the bollworm complex, pink bollworm (PBW), is one of the major damaging insect pests of cotton with an extensive range across India leading to severe loss to cotton production^7,8^.

Pink bollworm has become apparent as a threat to cotton cultivation in south and central cotton growing zones of India where the pest has developed resistance to Cry1Ac and Cry2Ab expressing cotton also developing resistance to insecticides and infesting late season cotton^8,9^. The PBW is assuming a major pest status even in some regions of northern India where there are ginning and oil extraction units which are procuring cotton seeds from central and south Indian cotton states where PBW has demonstrated resistance to Cry toxins in the field. So there is possibility of resistant pink bollworm infesting *Bt* cotton in North India through movement of seed. The evolution of resistance and pest adaptation to *Bt* crops containing Cry1Ac and Cry2Ab has been observed recently^10–12^. The development of resistance is due to multiple factors such as absence of refuge^13^ or supply of fraudulent refuge^13^, mono cropping, cultivation of long duration hybrids, extended cropping season^14^.

Bollgard-II (BGII) cotton expressing two proteins, Cry1Ac and Cry2Ab occupies approximately more than 90% of the area cultivating *G. hirsutum* cotton in India. BGII was expected to be effective against the pink bollworm especially after resistance to the single gene Cry1Ac was reported as heavy field infestations of PBW in Bollgard (BG), that was confined to Gujarat state in 2009^15^. Despite reports in 2015 of plausible breakdown of BGII resistance the contribution of stakeholders of the technology was grossly inadequate to ensure its sustainability^16^.

Pink bollworm (PBW) adaptation to transgenic *Bt-*cotton expressing Cry1Ac (BG) and ‘Cry1Ac + Cry2Ab’ (BGII) was assesed in 10 major cotton*-*cultivating states of India during 2010*–*2017. However the PBW larval incidence during this period on *Bt-*cotton was found less in north cotton cultivating zone of India, where as in central and south India, PBW larval recovery from BGII cotton bolls was high in the range of 28.85*–*72.49% during 2014*–*2017^8^. PBW infestation causes locule damage of 37.5% and 13.58% on non-*Bt* and *Bt* cotton respectively, at about 160 days of planting^17^. Presently PBW is assuming a major pest status even in northern India where it had minor pest status earlier. Recently the pink bollworm strain having 300-fold resistance to Cry1Ac, 2.6-fold cross-resistance to Cry2Ab identified and analyzed with novel cadherin allele (r16) builds its life cycle on transgenic *Bt* cotton containing Cry1Ac^18^.

Mitochondrial DNA is widely used in taxonomy and systematics to explore the phylogenetic relationships of insects^19,20^. Mitochondrial DNA (COI gene) is maternally inherited, well conserved and evolves in a nearly neutral fashion so it reflects the divergence times, which makes it a robust marker for determining genetic relationships and geographical studies^21–25^. Further the genetic constitution of a pest population is very vital in determining its capacity to tolerate adverse climatic conditions and adoption to new conditions^26^. Population genetic structure and genetic diversity defines the level of adaptation of a population to environmental change and susceptibility to selection pressure^27,28^. Gene flow through dispersion and migration which is responsible for determining genetic variation leads to evolution of local populations^29^. Even in some of the lepidopteran species, the genetic diversity and genetic structure are reported to be related to their migration capacity as well as number of generations^30,31^.

Studies on population structure and genetic diversity of PBW has been explored more in Asiatic countries such as India, Pakistan and China owing to the development of resistance to *Bt* cotton by PBW populations^32–34^. Liu *et al*.^35^ studied the population genetic structure of Chinese PBW using mitochondrial COII and Nad4 primers, and found extremely low genetic variability among all populations examined from nine provinces of China. Sequence variation in the Nad4 region differentiated the Chinese populations from the Pakistani and American populations. Haplotypes and differentiation in PBW populations of China was identified using piggyBac-like elements^33^. Sridhar *et al*.^34^, based on the analysis of pink bollworm population from 19 districts in 2011 and 2012 found that pink bollworm population in India exhibited low level of genetic diversity, and based on haplotype diversity results opined that the populations might be experiencing population expansion but could not provide the evidence through neutrality tests owing to the small population size.

Present investigation was designed to analyze the genetic diversity and distribution and to validate the theory of population expansion in different *Bt* resistant field collected Indian populations of *P. gossypiella* from three distinct cotton growing eco-zones of India spanning nine cotton growing states and forty four major cotton growing districts using mitochondrial cytochrome oxidase I (COI) gene. The study also aimed to understand the genetic diversity of populations of pink bollworm infesting early, collected from damaged rosette flowers and late season populations from infested bolls to understand the diversity of the two temporally separated populations of the cotton crop to attribute plausible reason for early outbreak of PBW.

## Materials and Methods

### Pink bollworm collection

**Figure 1:**
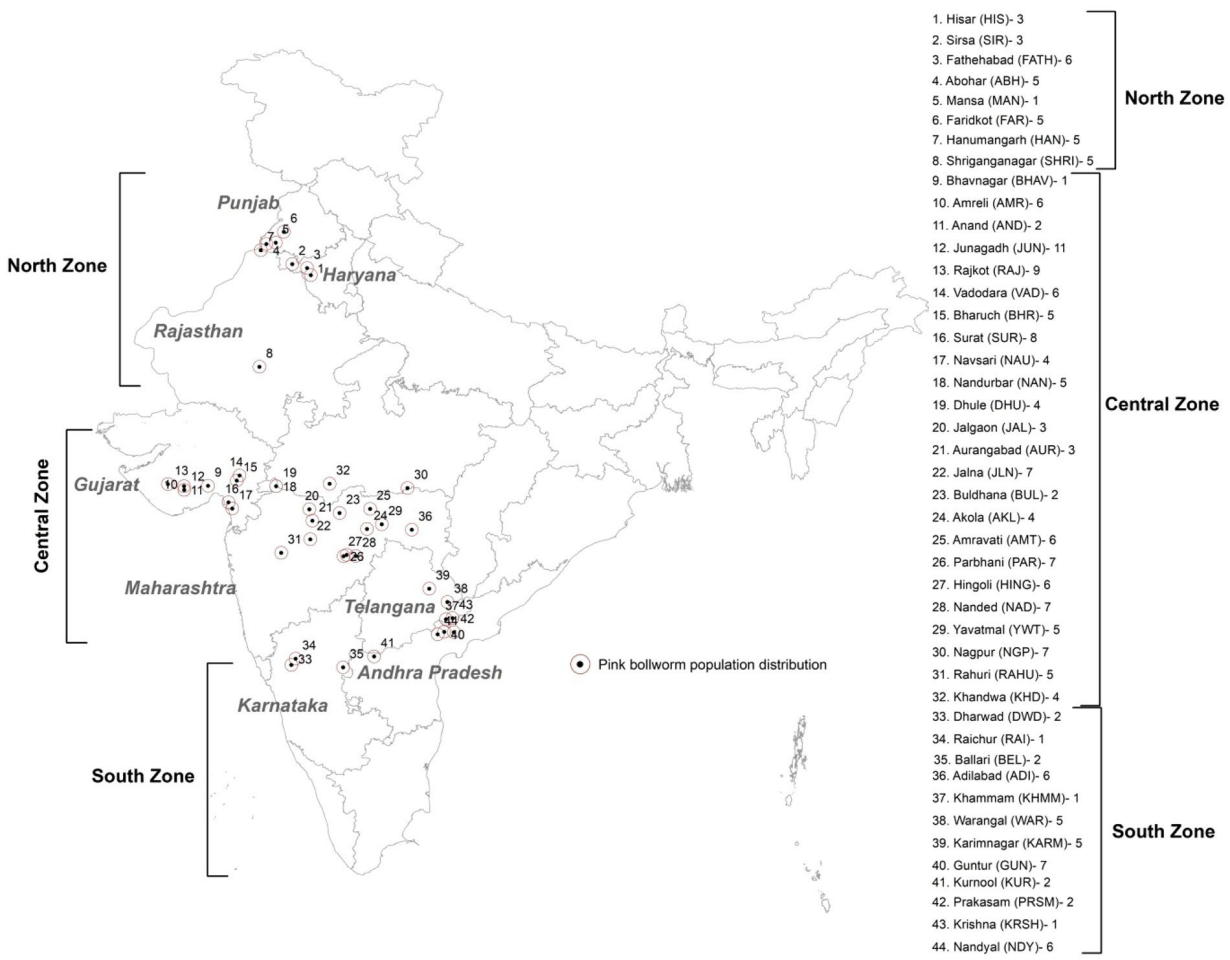
Sampling sites of *Pectinophora gossypiella* in India.

The larvae of *P. gossypiella* were collected from green bolls on cotton growing in different geographic locations of 44 districts of 9 states of India during 2017 and 2018 (Supplementary Table 1 & Fig. 1) for analysis of genetic diversity and to validate the theory of population expansion. To elucidate the nature of population during early and late season of the crop, the samples were collected from infested flowers as early population and infested bolls as late population from different plants as well as distant fields^36^.

### Extraction of total DNA

The third instar larvae were used for isolation of genomic DNA as per protocol standardized by Henry *et al*.^37^. Extracted genomic DNA was electrophoresed on 0.8% agarose gel to check the quality and quantification of DNA was done in spectrophotometer at A260/280 nm.

### PCR and sequencing of cytochrome oxidase I

Partial sequence of mitochondrial CO1 gene (850 bp) was amplified with universal primer COIF 5′TTGATTTTTTGGTCATCCAGAAGT3′ and COIR 5′TCCAATGCACTAATCTGCCATA TTA 3′ reported by Simonato *et al*., 2007. The total volume of PCR reaction of 25 µl mixture includes, template DNA(40 ng/µl) −2 µl, 10x taq buffer-2.5 µl, MgCl_2_(25 mM)−2 µl, dNTP(10 mM) −0.5 µl, forward primer (10 µM)-0.5 µl, reverse primer (10 µM)-0.5 µl, *Taq*DNA polymerase (3U)-0.4 µl and distilled water-16.6 µl. The PCR reaction was carried out in thermal cycler (Applied Bio systems) with the conditions such as initial denaturation at 94 °C for 5 min followed by 35 cycles of 94 °C for 45 s, primer annealing at 52 °C for 1 min and extension at 72 °C for 1 min with final extension at 72 °C for 5 min. The amplified products were resolved on 1.2% agarose gel, stained with ethidium bromide (10 mg/ml) and visualized in a gel documentation system (Biorad). Gel Extraction kit (QiagenVR) was used for extraction and purification of desired sized sample from agarose gel. After purification of samples from gel, samples were sequenced (both strands) through service of agrigenome Lab, Cochin, India using COI forward and reverse primer on automated ABI PRISM 3100 genetic analyzer (Applied Biosystems).

### Data analysis for genetic divergence and haplotype distribution

**Figure 2:**
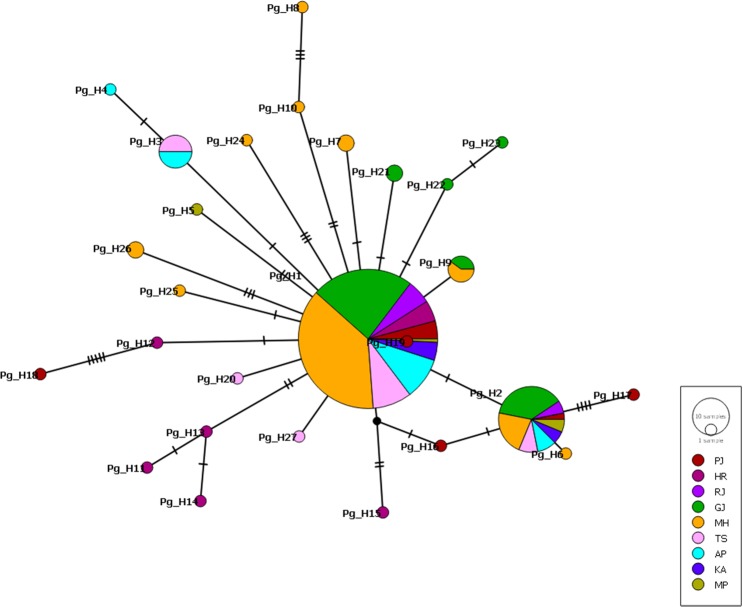
The TCS haplotype network tree for the mitochondrial COI region of PBW, circles represents the haplotypes identified and the size of the each circle are proportional to the frequency of the haplotypes. The lines between each haplotypes represents the mutations, each line represents single mutation.

Mitochondrial COI gene sequences were edited using Bio edit and aligned using ClustalW program in MEGA *ver*. 7.0.9 software^38^. Descriptive statistics number of haplotypes (H), haplotype diversity (Hd), variance and standard deviation of haplotype diversity were calculated using DnaSP *ver* 5.10.01 software^39^. To depict the evolutionary and geographical relationships among haplotypes, a median-joining (MJ) haplotype network was constructed with Propart *ver*. 1.7 software (Fig. 2). Genetic distances among zones were calculated based on pairwise matrix of sequence divergences using Kimura’s two parameter methods implemented in MEGA 7.0.9 software^40^.

### Phylogenetic analysis

**Figure 3:**
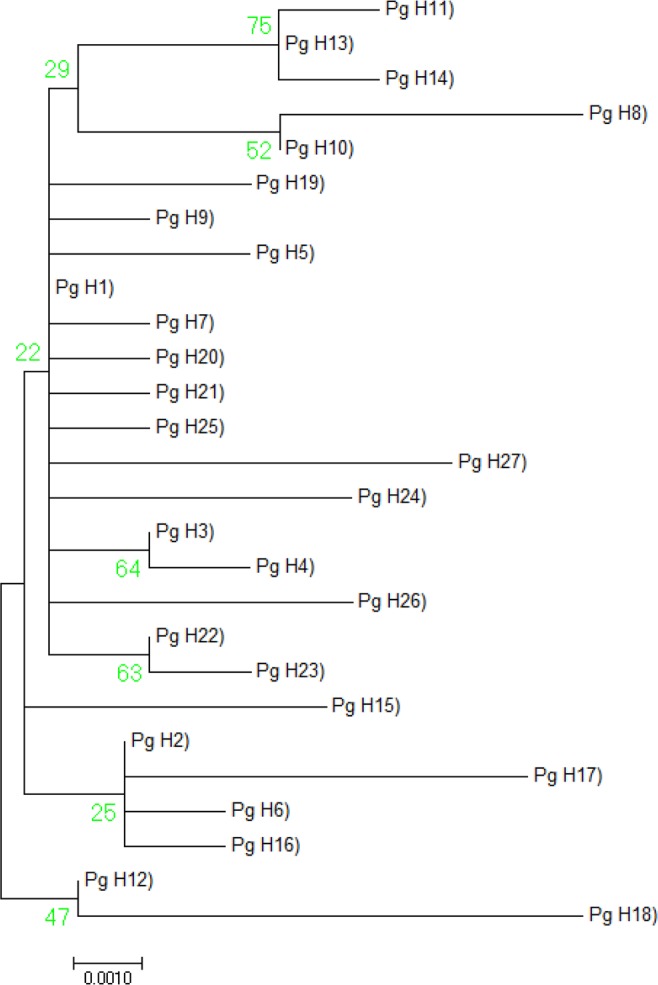
Phylogenetic tree of the 27 mtCOI DNA haplotypes in *Pectinophora gossypiella*.

The phylogenetic analysis based on the maximum likelihood (ML) method was performed using MEGA *ver*. 7.0.9 software for investigating the degree of consistency of mutation patterns in different regions of India. In these types of analyses, the nucleotide mismatch for each of region was selected using the Tamura–Nei model. The starting tree for ML was obtained via default neighbor-joining method and it is used for the ML heuristic method with very strong branch swap filter search (Fig. 3). The reliability of branches was assessed by 1000 bootstrap replications^41^.

### Neutrality test and genetic differentiation

Fu and Li’s D_test and F_test and Tajima’s D tests of neutrality index and genetic differentiation will be useful for demographic history information, with demographic expansion related to negative values and subdivided populations at equilibrium leading to positive values^42,43^ were also performed using DnaSP *ver*. 5.10.01 software for detecting the range of population expansions. The genetic differentiation (FST) between different state populations in range referring to the criterion by Wright^44^ defined genetic differentiation as low for FST < 0.05, moderate for 0.05 < FST < 0.15, high for 0.15 < FST < 0.25 and very high for FST > 0.25^45,46^. The goodness of fit of population expansion analysis was done with Raggedness indices model according to Harpending^47^.

## Results

### Evaluation of sequences from different regions and identification of haplotype and their distribution

To determine the genetic diversity and distribution, 686 bp trimmed nucleotide sequences of 214 mt-COI region represented by South (52), Central (129) & Northern (33) zones of three cotton-growing zones of India were used for analysis. The homology search of sequences using NCBI BLAST matched with the mt-COI sequences of pink bollworm in database, and the sequence similarity varied from 98–100% to that of available sequences of pink bollworm populations. The nucleotide sequences were aligned using MEGA *ver*. 7.0.9 and used for further analysis. The trimmed sequences were deposited in NCBI Gene Bank and accession numbers (MK652512-MK652704, MK775533-MK775550) were obtained.

The study identified a total of 27(12.61%) unique haplotypes in PBW populations of the three cotton growing zones of India (Fig. 3). Among the nucleotide mismatch or mutations found in different haplotypes, the transitional changes were more in number 21 (13 A = G, 8 T = C) while there were 16 transversions (5 A = C, 4 T = G and 7 A = T). Of these twenty seven, only eight haplotypes were shared by at least two populations. The most frequent haplotype (Pg_H1) was found in 143 individuals. The transition of base pair A to G was noticed in the Pg_H1 haplotype represented samples. Nearly 14.68, 62.23 and 23.07% of total individuals in north, central, and southern zone populations respectively were found to be Pg_H1 haplotype. The second most predominant haplotype (Pg_H2) noticed transition of base pair as G to A represented by 32 individuals and dominated populations represented central India similar to Pg_H1 haplotype (Table 1). The haplotype network was star-like and haplotypes were shared across different localities. Some haplotypes found in two or more sequences were (Pg_H9) haplotype was shared with Nanded_B4, Parbhani_B1, Parbhani_B5, Bharuch_F1 and Bharuch_F2 regions that belong to Maharashtra and Gujarat. Also haplotype (Pg_H3) was shared with populations of Guntur, Kurnool, Prakasam and Warangal region belonging to Andhra Pradesh and Telangana. The other 20 haplotypes (individual haplotypes) did not share any similarity with other populations.

**Table 1:** Haplotype distribution in different cotton growing zones of India.

Haplotype	North	Central	South	Total
Pg_H1	21	89	33	143
Pg_H2	3	21	8	32
Pg_H3			8	8
Pg_H4			1	1
Pg_H5		1		1
Pg_H6		1		1
Pg_H7		2		2
Pg_H8		1		1
Pg_H9		5		5
Pg_H10		1		1
Pg_H11	1			1
Pg_H12	1			1
Pg_H13	1			1
Pg_H14	1			1
Pg_H15	1			1
Pg_H16	1			1
Pg_H17	1			1
Pg_H18	1			1
Pg_H19	1			1
Pg_H20	1			1
Pg_H21		2		2
Pg_H22		1		1
Pg_H23		1		1
Pg_H24		1		1
Pg_H25		1		1
Pg_H26		2		2
Pg_H27			1	1
Total	34	129	51	214

### Genetic variation

In phylogenetic analysis of *P. gossypiella* the descriptive statistics Haplotype (gene) diversity (Hd), Variance of Haplotype diversity and Standard Deviation of Haplotype diversity were calculated with DnaSP *ver*. 5.10.01 software at 0.531, 0.00149 and 0.039, respectively, which suggest that entire population exhibited low level of genetic diversity. In this study zone-wise clustering revealed that central zone recorded low level of Hd (0.499) as compared to south (0.560) and north (0.595). Similarly, a state wise analysis revealed low levels of genetic diversity in Rajasthan (0.356) and Maharashtra (0.446) populations (Table 2).

**Table 2:** Haplotype and nucleotide diversity of Pink bollworm populations in India.

Location	n	K	Hd	pi	H
**Central India**	**129**	**0.00242**	**0.499**	**0.049**	**14**
Maharashtra	73	0.00504	0.446	0.071	10
Madhya Pradesh	4	0.04948	0.833	0.222	3
Gujarat	52	0.00450	0.526	0.067	6
**North India**	**33**	**0.00982**	**0.595**	**0.099**	**10**
Punjab	11	0.02084	0.727	0.144	6
Haryana	12	0.02196	0.682	0.148	6
Rajasthan	10	0.02532	0.356	0.159	2
**South India**	**52**	**0.00466**	**0.560**	**0.068**	**6**
Telangana	22	0.00989	0.623	0.099	5
Karnataka	8	0.02846	0.429	0.169	2
Andhra Pradesh	22	0.01076	0.567	0.104	4
**All**	**214**	**0.00149**	**0.531**	**0.039**	**27**

n:Number of samples; H:Number of haplotypes; Hd:Haplotypes diversity; K:Variance of haplotype diversity; pi:Standard deviation.

### Demographic history analysis

**Figure 4:**
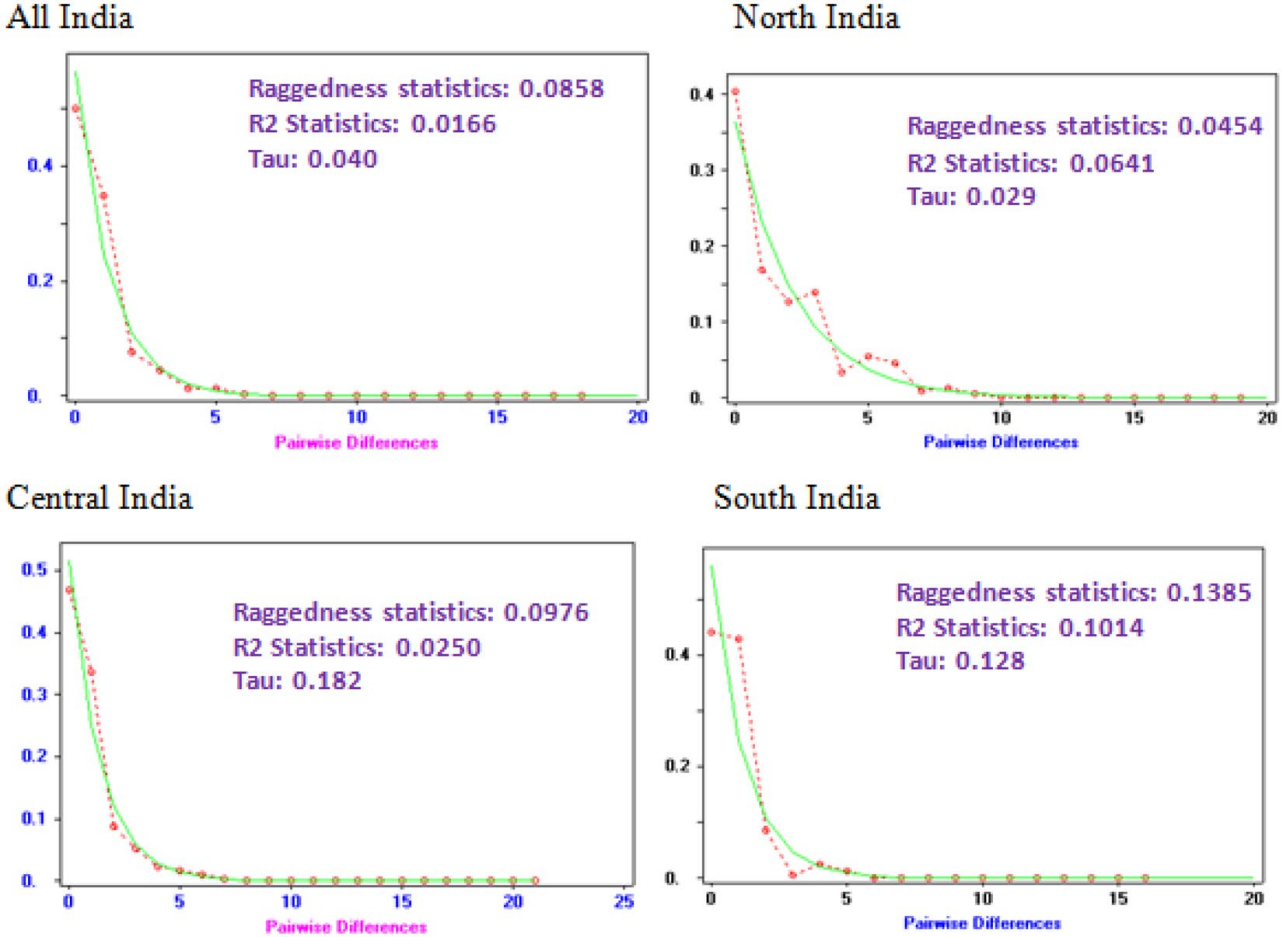
Observed and expected mismatch distributions for *Pectinophora gossypiella* in India, based on COI gene sequences for the mismatch distributions, the solid lines show observed frequency distribution while the dot lines show the distribution expected under the sudden-expansion model.

Tajima’s D test, Fu and Li’s D test and Fu and Li’s F neutrality tests were executed for analysis of demographic history in Indian pink bollworm populations. Neutrality tests were rejected for all populations with significant negative values which confers to the hypothesis of past population expansion events. The three neutrality tests were performed and values were negative for populations of India indicating there is an excess of rare mutations which favours population expansion or growth. As the observed mismatch distribution line which closely matched the expected line under a model of sudden expansion confirmed the results. The mismatch distribution plot which includes all-region populations was smooth and unimodal, indicating a population expansion, whereas the distribution of pairwise nucleotide differences (mismatch distribution) showed a slightly bimodal curve in north region while multimodal pattern in Haryana and Punjab region mismatch distributions may indicate strong population subdivision which confers a stable population size (Fig. 4). The raggedness index did not differ significantly from the expected under sudden and spatial population expansion models. These results obtained reject the hypothesis of neutral evolution for cotton PBW population from India (Table 3).

**Table 3:** Tajima’s D test, Fu and Li’s D test and Fu and Li’s F for COI gene in populations of Pink bollworm.

Location	Tajima’s *D*	Significance	Fu and Li’s *D*	Significance	Fu and Li’s *F*	Significance
**Central**	−2.21623	**, P < **0.01**	−2.95994	*, P < **0.05**	−3.20310	**, P < **0.02**
**North**	−2.14521	*, P < **0.05**	−2.82690	*, P < **0.05**	−3.06853	*, P < **0.05**
**South**	−1.61296	NS 0.10 > *p* > 0.05	−2.37273	NS 0.10 > *p* > 0.05	−2.50090	NS 0.10 > *p* > 0.05
**All**	−2.53885	**, P < **0.001**	−5.77307	**P < **0.02**	−5.25763	**P < **0.02**

### Genetic differentiation

The pairwise FST values ranged from −0.12018 to 0.10469. Of 28 comparisons, 9 showed moderate and the remaining 19 showed low genetic differentiation. The values of pairwise genetic distance between Gujarat population and other populations range from (−0.0359 to 0.10469) indicating low to moderate genetic differentiation.The low values (−0.01141 to 0.07145) were observed in Maharashtra population as compared to other populations. Comparison of Punjab populations with other populations revealed that genetic differences ranged from −0.00327 to 0.05875. Low to moderate values were observed in comparisons of the Haryana populations as compared to other populations (0.05875 to 0.10469) (Table 4).

**Table 4:** Comparison between populations Fst values.

	KAR	TEL	AP	RAJ	HAR	PUN	GUJ
**MAH**	−0.0062	0.02584	0.05176	−0.011	0.07152	0.01914	0.01695
**GUJ**	−0.0553	0.03309	0.06377	−0.0382	0.10469	0.00471	
**PUN**	−0.012	0.0241	0.03561	−0.0033	0.05875		
**HAR**	0.1013	0.08403	0.10456	0.09428			
**RAJ**	−0.1202	0.00961	0.0432				
**AP**	0.04029	−0.0295					
**TEL**	0.00893						

### Comparison of flower (early) and boll (late) populations of pink bollworm

**Figure 5:**
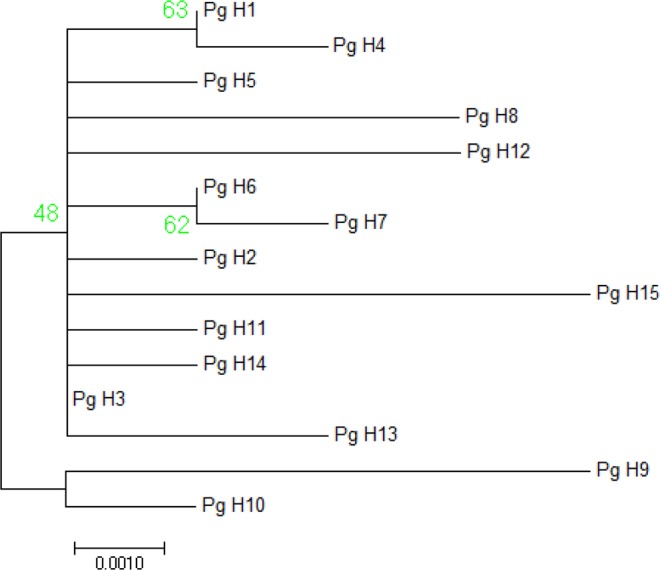
Phylogenetic tree of the 15mtCOI DNA haplotypes in the early and late populations of *Pectinophora gossypiella*.

The comparison of 36 early populations of Pink Bollworm which were collected from infested flowers was made with the same number of populations collected from infested bolls of 14 different locations (Table 5). Descriptive statistics Haplotype (gene) diversity (Hd), Variance of Haplotype diversity and Standard Deviation of Haplotype diversity with DnaSP ver. 5.10.01 were found to be 0.615, 0.00359 and 0.060 respectively. There are 15 different haplotypes that were observed (Fig. 5). Haplotype 2 represented due to transition of G to A nucleotide was observed to be dominant for the late season PBW populations (12) collected from infested boll except in one population collected from infested flower. Haplotype 3 due to transition of A to G nucleotide was present in 43 populations dominated by 27 early season PBW populations. Haplotype 10 was shared with 3 late season populations from Nanded and Parbhani districts. The values of pairwise genetic distance between early and late populations found 0.08438 indicated moderate genetic differentiation. Rejection of Neutrality tests Fu and Li’s D, Fu and Li’s F and Tajima’s D with significant negative values indicated the demographic expansion. It must be mentioned that early and late season populations were morphologically similar as adults.

**Table 5:** The geographic locations for comparison of early and late season populations of PBW.

Sr.no.	State	Locations	Collection ID	No. of Boll sequence	No. of flower sequence
**1**.	Punjab	Faridkot	FAR	1	1
**2**.	Gujarat	Amreli	AMR	2	2
**3**.	Gujarat	Junagadh	JUN	3	3
**4**.	Gujarat	Rajkot	RAJ	3	3
**5**.	Gujarat	Vadodara	VAD	3	3
**6**.	Gujarat	Bharuch	BHR	1	1
**7**.	Gujarat	Surat	SUR	3	3
**8**.	Maharashtra	Jalna	JLN	3	3
**9**.	Maharashtra	Parbhani	PAR	3	3
**10**.	Maharashtra	Nanded	NAD	3	3
**11**.	Maharashtra	Yavatmal	YWT	1	1
**12**.	Maharashtra	Nagpur	NGP	3	3
**13**.	Telangana	Adilabad	ADI	3	3
**14**.	Andhrapradesh	Guntur	GUN	4	4
				36	36

## Discussion

The objective of this study was to firstly identify the different haplotypes of P. *gossypiella*, major pest of cotton and secondly to see its distribution in the different agro-ecological zones and in the sampled localities of India in light of its occurrence and resistance development to *Bt* cotton. After a careful alignment of the 214 COI mitochondrial gene sequences, we have investigated the genetic diversity and structure of 214 individuals of 44 populations sampled throughout their main area of distribution in India. The mitochondrial DNA sequence region was used in this study because it is more prone to genetic drift than nuclear markers and because of the smaller effective population size and maternal gene flow^35,48,49^.

The studies revealed that total of 27 unique haplotypes were identified in 214 individuals from all over India, with low values of nucleotide and haplotype diversity and is supported by Sridhar *et al*.^34^ where they found 12 (15.18%) haplotypes in 79 individuals distributed in 19 populations of Indian subcontinent. The eight haplotypes were shared by at least two populations other 19 haplotypes were unique and did not share the same ancestral haplotype. The haplotype (Pg_H1) was most distributed as it was found in 143 individuals, and the second most predominant haplotype (Pg_H2) was shared with 32 individuals as confirmed with the star-like haplotype network shared by populations across different zones (Fig. 2).

Accurate assimilation and understanding of the genetic diversity of an insect pest is found essential to mitigate and improve its monitoring that further facilitates implementation of need based independent strategies. In this study zone-wise clustering revealed that the central zone recorded low level of Hd as compared to south and north zones. Low mitochondrial DNA variations are reported in taxa that might have undergone severe bottlenecks or founder effects^49–51^. These results are supported by Sridhar and co-workers^34^ who found 0.3028 Hd in overall populations with lowest in central zone (0.2730) in 79 adult individual moths during 2011–2012 populations of pink bollworm before full blown resistance to both Cry1Ac and Cry2Ab in India. Similarly extremely low level of population genetic variation was observed in the two mitochondrial regions (COII and Nad4) among the nine Chinese Pink Bollworm populations, with only one to four mitochondrial haplotypes which were attributed to invasion bottlenecks, which had subsequently strengthened by its non-migratory biology and the mosaic pattern of agricultural activities^32^.

Commercialization of *Bt* cotton might have influenced the evolutionary status of PBW as it is having narrow host range and limited dispersal ability^52,53^. The non-migratory behavior might also have influenced the genetic variation as dispersal patterns influence genetic variation in Lepidoptera species due to limited gene flow among populations^54,55^. To control broader range of pests *Bt* crops expressing new genes and multiple genes can be developed also in some countries like United States and Australia the non *Bt* cotton refuge strategy in which 25% of the area planted to cotton with a single *Bt* toxin protein as this area can be reduced by using two *Bt* proteins^56–58^. The high*-*dose refuge management strategy in which mating between susceptible and resistant individuals has been key to postpone counteract evolution of *Bt* resistance^59^.

The current distribution of mt-DNA haplotypes in PBW seems highly indicative of demographic expansion in Indian populations. Significantly negative departures from zero for neutrality tests values also support population expansions^42,60,61^. Overall, the haplotype network showed a star-shaped cartography as characteristic of population expansion after a bottleneck, wherein newer mutations form groups of lower-frequency haplotypes budding from a central haplotype (Fig. 2). Neutrality tests, distributions of pairwise differences (mismatch distributions) obtained with COI gene data from the overall populations are found unimodal, suggesting that the populations of *P. gossypiella* experience population expansion. The star shape of the haplotype network with the existence of multiple haplotypes support hypothesis of expansion. Considering the presence of an ancestral haplotype and less isolation-by-distance relationships of this species, we can conclude that *P. gossypiella* in India has not experienced parallel evolutions.

## Conclusions

We investigated the population genetic diversity and structure of 214 populations and haplotype distribution of *P. gossypiella* in India sampled throughout 44 geographical populations. The results revealed low genetic diversity of *P. gossypiella* in sampled areas. Phylogenetic analysis of the mitochondrial gene haplotypes identified two ancestral haplotypes present in India. Our research provides a successful example of a method for understanding the seasonal movement of insects. Significantly negative departures from zero for Tajima’s *D*, Fu and Li’s D test and Fu and Li’s F neutrality tests values also support population expansions and the distributions of pairwise differences (mismatch distributions) obtained with COI gene data. Similar trend was observed in early and late season populations of PBW. Genetic data thus obtain not only provides us with an perceptive of population genetics, but also that the two populations of pink bollworm, those occurring early in the season is genetically close to the late season populations with respect to their CO1 region.

## Supplementary information

Supplementary Information.
