# Supplementary material for: Evidence for population expansion of Cotton pink bollworm Pectinophora gossypiella (Saunders) (Lepidoptera: Gelechiidae) in India
Source: Sci Rep. 2020 Mar 16;10:4740. doi: 10.1038/s41598-020-61389-1 (PMC7075961; doi:10.1038/s41598-020-61389-1)
Supplement: Supplementary file 1 — Supplementary Information. [file 41598_2020_61389_MOESM1_ESM.docx]

**Evidence for population expansion of Cotton pink bollworm *Pectinophora gossypiella* (Saunders) (Lepidoptera: Gelechiidae) in India**

V. ChinnaBabu Naik^a*^, Pratik P. Pusadkar^a^, Sandesh T. Waghmare^a^, Raghavendra K. P^a^, Sandhya Kranthi^a^, Sujit Kumbhare^a^, V. S. Nagrare^a^, Rishi Kumar^b^, Tenguri Prabhulinga^a^, Nandini Gokte-Narkhedkar^a^, V. N. Waghmare^a^

^a^ ICAR-Central Institute for Cotton Research. Nagpur, Maharashtra, India.

^b^ ICAR-Central Institute for Cotton Research, Regional station, Sirsa

* Corresponding author: Crop Protection Division, Central Institute for Cotton Research, Post Bag No.2, Shankar Nagar P.O, Nagpur 440010, Maharashtra, India. e-mail: chinnaenton@gmail.com

**Supplementary Table 1**.**Details of geographic locations of Pink bollworm collection**

| **S.n.** | **State** | **Locations** | **Collection ID** | **Sample size** | **Date of Collection** | **Latitude N** | **Longitude E** | **Gene bank number** |
| --- | --- | --- | --- | --- | --- | --- | --- | --- |
| **1** | Haryana | Hisar | HIS | 3 | 13/10/2017 | 29°09'01’36'' | 75°41'33’58'' | MK652594-MK652596 |
| **2** | Haryana | Sirsa | SIR | 3 | 15/10/2018 | 29°42'62’29'' | 75°09'50’40'' | MK652597-MK652599 |
| **3** | Haryana | Fatehabad | FATH | 6 | 13/08/2017 | 29°23'52’68'' | 75°33'51’98'' | MK652591-MK652593 |
| **4** | Punjab | Abohar | ABH | 5 | 15/10/2017 | 30°14'37’46'' | 74°07'55’93'' | MK652600-MK652604 |
| **5** | Punjab | Mansa | MAN | 1 | 15/10/2017 | 30°17'21’40'' | 74°27'29’09'' | MK652610 |
| **6** | Punjab | Faridkot | FAR | 5 | 14/10/2017 | 30°40'25’81'' | 74°44'41’41'' | MK652605-MK652609 |
| **7** | Rajasthan | Hanumangarh | HAN | 5 | 15/10/2017 | 30°01'36’66'' | 73°55'59’17'' | MK652611-MK652615 |
| **8** | Rajasthan | Sriganga  nagar | SHRI | 5 | 14/10/2017 | 25°55'52’62'' | 73°53'19’92'' | MK652616-MK652620 |
| **9** | Gujarat | Bhavnagar | BHAV | 1 | 11/01/2018 | 21°45'06'53'' | 72°04'52'13'' | MK652516 |
| **10** | Gujarat | Amreli | AMR | 6 | 12/01/2018 | 21°36'25'46'' | 71°14'32'79'' | MK652512-MK652513, MK652650-MK652653 |
| **11** | Gujarat | Anand | AND | 2 | 12/01/2018 | 21°44'20'13'' | 71°14'09'33'' | MK652513, MK652514 |
| **12** | Gujarat | Junagadh | JUN | 11 | 12/01/2018 | 21°36'25'46'' | 71°14'32'79'' | MK652517, MK652518,  MK652519, MK652658-  MK652665 |
| **13** | Gujarat | Rajkot | RAJ | 9 | 12/01/2018 | 31°48'36'22'' | 70°40'01'33'' | MK652524, MK652525  MK652526, MK652666  -MK652671 |
| **14** | Gujarat | Vadodara | VAD | 6 | 13/01/2018 | 22°06'56'38'' | 73°11'24'33'' | MK652530, MK652531  MK652532, MK652677  -MK652679 |
| **15** | Gujarat | Bharuch | BHR | 5 | 13/01/2018 | 21°56'03'52'' | 73°05'07'38'' | MK652515, MK652654  -MK652657 |
| **16** | Gujarat | Surat | SUR | 8 | 13/01/2018 | 21°10'14'02'' | 72°47'56'11'' | MK652527-MK652529, MK652672-MK652676 |
| **17** | Gujarat | Navsari | NAU | 4 | 13/01/2018 | 20°56'60'00'' | 72°55'48'02'' | MK652520-MK652523 |
| **18** | Maharashtra | Nandurbar | NAN | 5 | 24/11/2017 | 21°43'60'71'' | 74°28'12'05” | MK652574-MK652578 |
| **19** | Maharashtra | Dhule | DHU | 4 | 24/11/2017 | 20°55'47'49'' | 74°51'02''91” | MK652552-MK652555 |
| **20** | Maharashtra | Jalgaon | JAL | 3 | 24/11/2017 | 20°55'33'09'' | 75°38'29'64'' | MK652562- MK652564 |
| **21** | Maharashtra | Aurangabad | AUR | 3 | 27/11/2017 | 20°31'19'32'' | 75°44'48'05'' | MK652547-MK652549 |
| **22** | Maharashtra | Jalna | JLN | 7 | 25/11/2017 | 19°52'13'40'' | 75°40'27'17'' | MK652565-MK652567, MK652687,MK652688, MK652680, MK652681 |
| **23** | Maharashtra | Buldhana | BUL | 2 | 27/11/2017 | 20°47'35'23'' | 76°42'10'56'' | MK652550, MK652551 |
| **24** | Maharashtra | Akola | AKL | 4 | 27/11/2017 | 20°14'07'04'' | 77°40'05'21'' | MK652537-MK652540 |
| **25** | Maharashtra | Amravati | AMT | 6 | 27/11/2017 | 20°56'14’72'' | 77°46'46’37'' | MK652541-MK652546 |
| **26** | Maharashtra | Parbhani | PAR | 7 | 22/12/2017 | 19°16'27'46'' | 76°50'23'74” | MK652579-MK652581, MK652692-MK652695 |
| **27** | Maharashtra | Hingoli | HING | 6 | 24/12/2017 | 19°18'85'29'' | 76°57'08'87” | MK652556-MK652561 |
| **28** | Maharashtra | Nanded | NAD | 7 | 25/12/2017 | 19°16'50'01'' | 77°16'08'63'' | MK652570-MK652573  MK652689-MK652691 |
| **29** | Maharashtra | Yavatmal | YWT | 5 | 23/12/2017 | 20°23'46'16'' | 78°11'15'15'' | MK652587-MK652590, MK652696 |
| **30** | Maharashtra | Nagpur | NGP | 7 | 6/12/2017 | 21°40'16'63'' | 79°05'19’89'' | MK652568, MK652569, MK652682-MK652686 |
| **31** | Maharashtra | Rahuri | RAHU | 7 | 22/11/2017 | 19°23'33.63'' | 74°38'55.77'' | MK652582-MK652586 MK775549 MK775550 |
| **32** | Madhyapradeh | Khandwa | KHD | 4 | 24/11/2017 | 21°49'32.6388'' | 76° 21' 9.25'' | MK652533-MK652536 |
| **33** | Karnataka | Dharwad | DWD | 2 | 25/11/2017 | 15°2736.9072'' | 75° 0' 37.02'' | MK652633-MK652634 |
| **34** | Karnataka | Raichur | RAI | 4 | 25/11/2017 | 15°40'32.6082'' | 75°09'27.15'' | MK652635  MK775533-MK775535 |
| **35** | Karnataka | Ballari | BEL | 2 | 25/11/2017 | 15°08'21"08 | 76°55'12.04" | MK775536, MK775537 |
| **36** | Telangana | Adilabad | ADI | 6 | 18/11/2017 | 19°72'20'46'' | 78°74'28'36'' | MK652636-MK652638, MK652701-MK652703 |
| **37** | Telangana | Khammam | KHMM | 1 | 17/11/2018 | 17°03'30'56'' | 80°25'77'44'' | MK652644 |
| **38** | Telangana | Warangal | WAR | 10 | 17/11/2017 | 17°40'07'36'' | 79°89'39'06'' | MK652645-MK652649 |
| **39** | Telangana | Karimnagar | KARM | 5 | 17/11/2017 | 18°07'78'27'' | 79°50'81'07'' | MK652639-MK652643 |
| **40** | Andhrapradesh | Guntur | GUN | 10 | 16/11/2017 | 16°37'5'01'' | 80°23'01'19'' | MK652621-MK652623, MK652697-MK652700, MK775538-MK775540 |
| **41** | Andhrapradesh | Kurnool | KUR | 2 | 15/11/2017 | 16°72'71.69'' | 76°93'08’42'' | MK775547, MK775548 |
| **42** | Andhrapradesh | Prakasam | PRSM | 3 | 16/11/2017 | 15°95'84'18'' | 80°42'69'02'' | MK652631-MK652632 |
| **43** | Andhrapradesh | Krishna | KRSH | 1 | 16/11/2017 | 16°66'12'24'' | 80°40'25'07'' | MK652624 |
| **44** | Andhrapradesh | Nandyal | NDY | 6 | 16/11/2017 | 16°32'08'04'' | 80°08'69'17'' | MK652625-MK652630 |
